# Seroprevalence of Cystic Echinococcosis in Yaks and Sheep During 2017 on the Qinghai–Tibet Plateau, China

**DOI:** 10.3389/fvets.2022.849500

**Published:** 2022-03-24

**Authors:** Xing Gao, Luosong Xire, Zhao Zhang, Chuxian Quan, Shimeng Zhou, Kewei Li, Rende Song, Suonan Zhao, Xiangying Kong, Cairang Naori, Muhammad Fakhar-e-Alam Kulyar, Yuhua Bao, Jiakui Li

**Affiliations:** ^1^College of Veterinary Medicine, Huazhong Agricultural University, Wuhan, China; ^2^Veterinary Biological Medicine Manufacturing Factory of Tibet Autonomous Region, Lhasa, China; ^3^Qinghai Animal and Veterinary Sciences Work Station, Yushu, China; ^4^Haibei Agricultural and Animal Husbandry Sciences Institute, Haibei, China; ^5^Animal Husbandry and Veterinary Science Research Institute of Gannan Prefecture, Gannan, China; ^6^College of Animals Husbandry and Veterinary Medicine, Tibet Agricultural and Animal Husbandry University, Linzhi, China

**Keywords:** seroprevalence, *Echinococcus granulosus*, risk factors, yaks, Tibetan sheep

## Abstract

Cystic echinococcosis (CE) is a livestock disease caused by a parasite known as *Echinococcus granulosus*. It is one of the primary cause for illness and poverty especially for herders on the Qinghai–Tibet plateau, China. Meanwhile, the Qinghai–Tibet plateau has been a key area for echinococcosis control in China. Here in current study, we determined the seroprevalence of *E. granulosus* in ruminants on this region. A total of 2,730 serum samples (1,638 samples from yaks and 1,092 samples from sheep) were collected on the plateau during the period of 2017. The samples were assayed for *E. granulosus* antibodies by commercial enzyme-linked immunosorbent assay kits. Our results exhibited a prevalence percentage of 52.2% in Tibetan yaks and 38.2% in Tibetan sheep. Moreover, there was more chance of being infected with *E. granulosus* infection in old animals due to more exposure to contaminated sources of infection. However, no significant difference was observed. Furthermore, we observed that the rainfall and presence of several lakes has increased the risk of CE infection in yaks and sheep in the Qinghai, Qinglong, and Baingoin areas. Hence, with this investigation, it was possible to determine the frequency and distribution of CE in yaks and Tibetan sheep on the Qinghai-Tibet plateau, that laying the groundwork for its prevention and management.

## Introduction

Cystic echinococcosis (CE) is a livestock disease caused by a parasite called *Echinococcus granulosus*. It is transmitted by dogs, wolves, and foxes, causing different symptoms in different viscera or brain (1–3). Approximately 30 million livestock are infected with this globally distributed disease every year, causing more than 1.92 billion US dollars loss to the global animal husbandry (4, 5). Moreover, the health of livestock and herders is seriously endangered with the low development of breeding industry under the affect of CE. It is one of the main factors causing illness and making herders poor on the Qinghai–Tibet plateau. The Qinghai–Tibet plateau has been a key area for echinococcosis control in China.

*E. granulosus* is mainly found in low-lying moist areas and swamps (6, 7). The strategies to control the risk of *E. granulosus* are more important particularly in such areas, where humans and domestic livestock are in the same environment (8–12).

A number of diagnostic tests are available for the detection of *E. granulosus*, such as polymerase chain reaction, enzyme-linked immunosorbent assay (ELISA), indirect ELISA, and colloidal gold method (13–18). The ELISA approach is notable for its inexpensive cost, increased sensitivity, and specificity as compared to other methods, which often overlook infections with low parasitemia (14, 15).

In current research, we determined the seroprevalence of *E. granulosus* in ruminants using ELISA. With the investigation, the prevalence and distribution of CE were basically clarified in yaks and Tibetan sheep on the Qinghai–Tibet plateau, which provided a basis for the prevention and control of the disease.

## Materials and Methods

### Information of Collecting Region

The Qinghai–Tibet plateau is located on the southwestern border of China and south-central Eurasia. It is the largest and highest plateau in China (latitude and longitude, 20°00'−39°47'N and 73°19'−104°47'E, respectively). The average altitude is above 4,000 m with a complex climate, low temperature, and a sufficient sunshine. There were more than 300 lakes within 10 km^2^ on the plateau. Also, it is one of the important pastoral areas in China with abundant grassland (19).

### Information of Sampled Animals

Yak is a unique bovine species on the Qinghai–Tibet plateau. More than 14 million yaks are mainly distributed on the Qinghai–Tibet plateau in China, while there is a small distribution in Afghanistan, India, and Pakistan. The yaks are necessary for herders because of the milk, wool, and meat (20, 21). Tibetan sheep is one of the three original varieties in China and the biggest proportion in livestock, with more than 30,000,000 sheep on the plateau (22).

### Serum Samples

**Figure 1:**
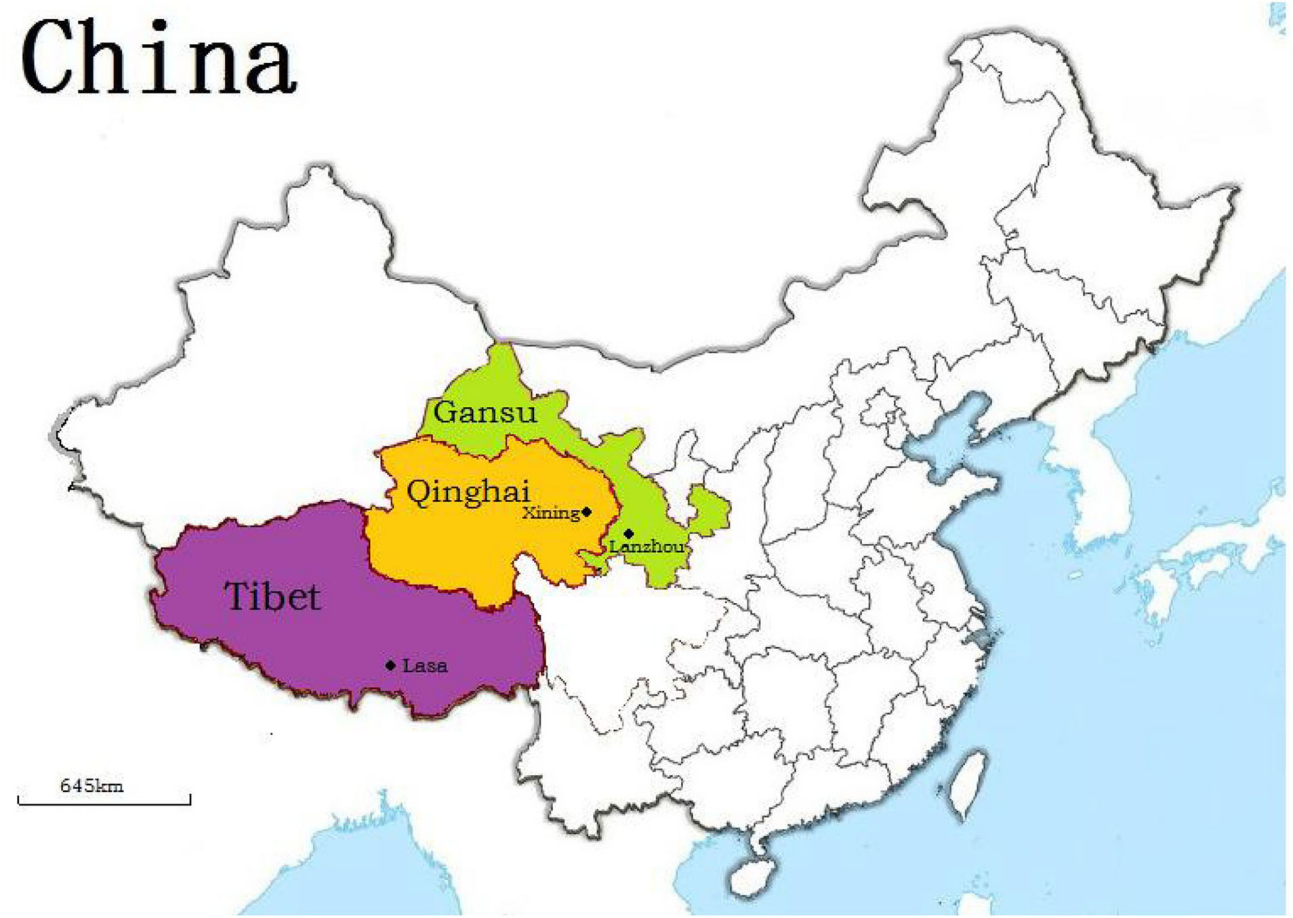
Geographic distribution of yaks enrolled in study.

**Figure 2:**
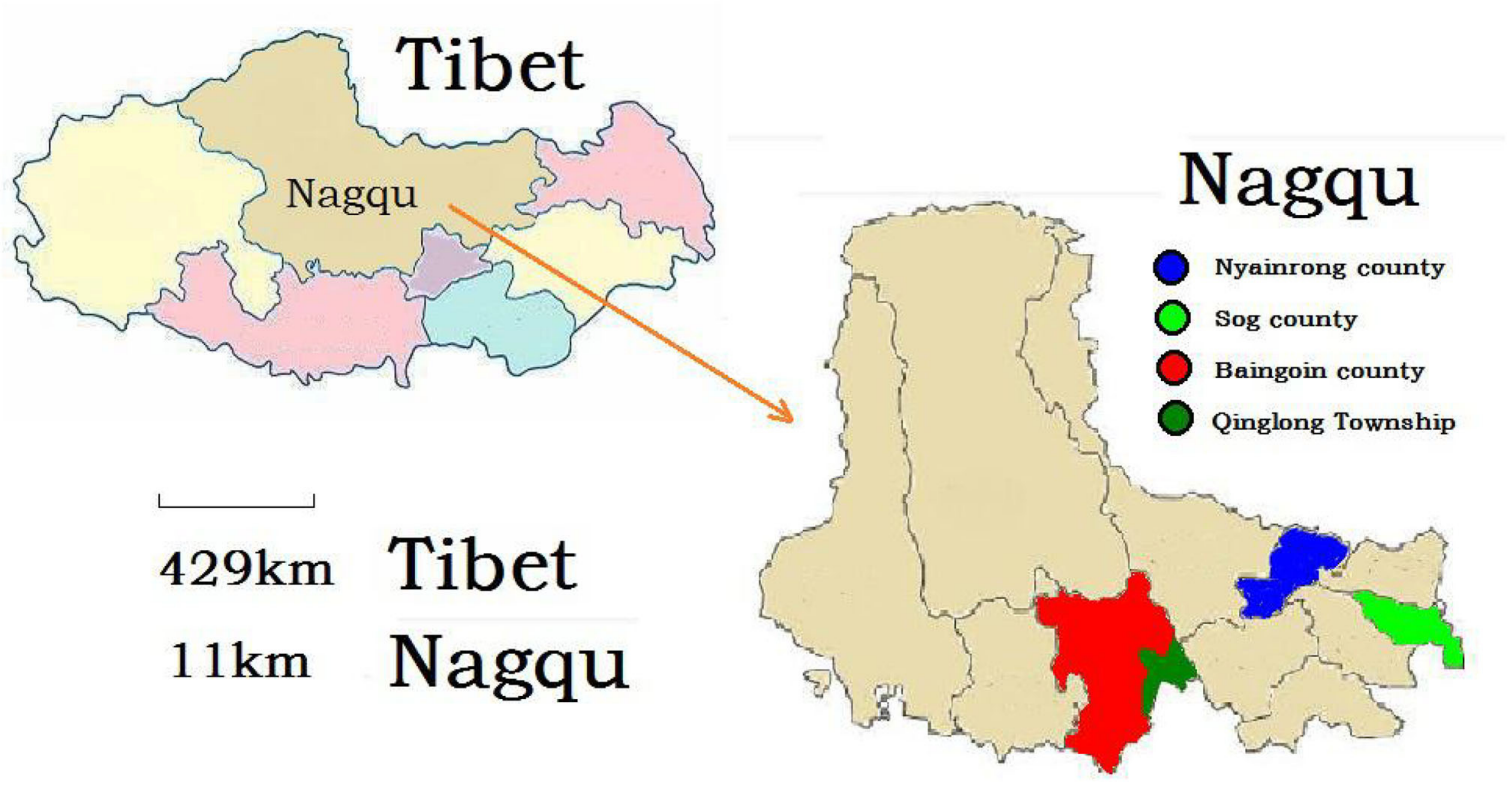
Geographic distribution of Tibetan sheep enrolled in study.

A total of 2,370 blood samples (1,638 samples from yaks, Figure 1; 1,092 samples from sheep, Figure 2) were collected during 2017 on Qinghai, Gansu, and Tibet, respectively. Age, sex, and region was the information that obtained for each animal, involving this study. Then serum of each animal was separated by centrifugation and stored at −20°C till analysis.

### Determination of Antibodies Against *E. granulosus*

All serum had been determined for anti-*E. granulosus* antibodies by using two commercial enzyme-linked immunosorbent kits (Jianlun Biological Pharmaceuticals Co., Ltd., Guangzhou China; Duoyu Biological Pharmaceuticals Co., Ltd., Shanghai, China) according to the manufacturer's instructions. The detailed method was consistent with the previous research (23).

## Results

A total of 855 of the 1,638 (52.2%) yaks were detected to have a CE infection, of which 328 (50.2%) were males and 527 (53.6%) were females. The prevalence values were 45.1, 64.3, and 49.5% on Tibet, Qinghai, and Gansu, respectively. While the prevalence ranged from 17.4 to 57.5% in different ages (Table 1).

**Table 1:** Prevalence and risk factors of *Echinococcus granulosus* infection in yaks on Qinghai–Tibet plateau.

**Variable**	**Category**	**No. tested**	**No. positive**	**% (95% CI)**	***P*-value**	**OR (95% CI)**
Region	Tibet	819	369	45.1 (41.6–48.5)	Reference	
	Qinghai	546	351	64.3 (60.1–68.3)	<0.001	2.20 (1.36–2.75)
	Gansu	273	135	49.5 (43.4–55.5)	0.207	1.19 (0.64–1.92)
Gender	Male	654	328	50.2 (43.6–54.1)	Reference	
	Female	984	527	53.6 (50.4–56.7)	0.177	1.15 (0.72–1.56)
Age	0 < year ≤ 1	109	19	17.4 (10.8–25.9)	Reference	
	1 < year ≤ 2	90	38	42.2 (31.9–53.1)	<0.001	3.46 (1.15–5.55)
	2 < year ≤ 4	570	298	52.3 (48.1–56.4)	<0.001	5.19 (4.11–6.32)
	Year > 4	869	500	57.5 (54.2–60.9)	<0.001	6.42 (4.09–8.26)
Total		1,638	855	52.2 (49.7–54.6)		

In the current research, the more influencing risk factors were region and age according to logistic regression models. Qinghai yaks were considered to be 2.20 times of higher risk of being positive compared with Tibet yaks, whereas Gansu yaks were considered to be 1.19 times at higher risk of CE infection compared with Tibet yaks (Table 1). In different ages, yaks <1 year ≤ 2 years had 3.46 times higher risk of CE infection compared with yaks <0 years ≤ 1 year; both yaks of <2 years ≤ 4 years and >4 years (57.54%) had 5.19 times and 6.42 times higher risk of being positive, respectively, when compared with yaks <0 years ≤ 1 year (Table 1). Also, there was no significant difference between males and females for yaks (Table 1).

In our research, 1,092 serum samples of Tibetan sheep were tested, 38.2% were detected to be positive for *E. granulosus*, with the distribution of 25.3% (Sog county), 15.4% (Nyainrong), 54.4% (Qinglong), and 56.8% (Baingoin) (Table 2). Tibetan sheep from both Nyainrong and Sog county had a significantly lower risk of CE infection compared with that from Qinglong and Baingoin (Table 2). With regard to sex, there was a non-significant difference. However, the seroprevalence values were 25.7% (juveniles), 42.8% (sub-adults), and 50.7% (adults) (Table 2). The Tibetan sheep in sub-adults and adults had two times higher risk of CE infection in juveniles (Table 2).

**Table 2:** Prevalence and risk factors of *Echinococcus granulosus* infection in Tibetan sheep on Qinghai–Tibet plateau.

**Variable**	**Category**	**No. tested**	**No. positive**	**% (95% CI)**	***P*-value**	**OR (95% CI)**
Region	Sog County	273	69	25.3 (20.2–30.9)	0.004	1.86 (0.35–3.82)
	Nyainrong County	273	42	15.4 (11.3–20.2)	Reference	
	Qinglong Township	171	93	54.4 (46.6–62.0)	<0.001	6.56 (5.10–7.24)
	Baingoin County	375	213	56.8 (51.6–61.9)	<0.001	7.23 (6.09–8.20)
Gender	Male	616	249	40.4 (36.5–44.4)	Reference	
	Female	476	168	35.3 (31.0–39.8)	0.084	1.24 (0.97–1.59)
Age	0 < year ≤ 1	393	101	25.7 (21.4–30.3)	Reference	
	1 < year ≤ 2	484	207	42.8 (38.3–47.3)	<0.001	2.16 (2.35–2.62)
	Year > 2	215	109	50.7 (43.8–57.6)	<0.001	2.97 (2.24–3.48)
Total		1,092	417	38.2 (35.3–41.1)		

## Discussion

Parasites have lived on Earth for as long as life has existed, and no species, whether animal or human, is exempt to parasites (24). *E. granulosus* had caused a tremendous economic loss and a severe public health risk in China as a foodborne neglected parasitic disease (25). More than seven million livestock are infected by CE yearly (8).

The seroprevalence of CE infection in yaks was 52.2% in our study, which was higher than the prevalence in the previous research in Turkey (41.1%), Greece (42%), and Ethiopia (27.6%), and significantly higher than the prevalence in Southern Brazil (13.7%) and Pakistan (13.46%) (3). Meanwhile, the previous investigation showed that the average infection rates of sheep decreased from 8.17% in 2012 to 3.68% by 2018 in the Western Sichuan Plateau (26). It was also significantly lower than the seroprevalence of CE infection in Tibetan sheep in our research (38.2%).

The previous study identified that the prevalence of CE was bound with environment culture, exerting complicated and combined effects (27). Meanwhile, the low temperatures could be a possible reason for the seroprevalence of CE. The Qinghai–Tibet plateau had a low surface temperature for years and has been a suitable place for CE due to its high altitude (28, 29). On the other hand, the stagnant economy is one of the main reason for CE. In rural areas, people had no awareness to undergo a personal medical checkup before the illness. People could be infected easily by echinococcosis due to the lack of education, information, and low sanitation conditions (30–33). A recent study showed that the plateau is the key area of CE in China (3).

In our research, the analysis showed that *E. granulosus* infection is closely related to region and age of animals. Due to the rainfall and many lakes, the ruminants had a higher risk of CE infection in Qinghai province, Qinglong, and Baingoin (23). In addition, the results suggested that old animals had more chances to acquire *E. granulosus* infection due to more exposure to the source of infection. However, no significant difference was observed in sex. Therefore, persons had a much higher risk of *E. granulosus* infection by frequent exposure to infected animals.

## Conclusion

Our research showed the high seroprevalence rate of *E. granulosus* infection in ruminants on the Qinghai–Tibet plateau in China. It was indicated that *E. granulosus* could cross-transmit between the environment and host, including human beings. Therefore, effective measures must be taken to control the spread of *E. granulosus* by considering the role of various factors. Hence, our study might be useful to wiping off such transmissible disease on Qinghai–Tibet plateau of China.

## Data Availability Statement

The original contributions presented in the study are included in the article/supplementary material, further inquiries can be directed to the corresponding author/s.

## Ethics Statement

Blood samples were collected under the permission of the relevant institutions. All procedures were approved and performed by Laboratory Animals Research Centre of Hubei, Qinghai, Gansu and Tibet in China, and the Ethics Committee of Huazhong Agricultural University, China (Permit number: 4200695757). All animal experiments and procedures were conducted under the relevant procedures of Proclamation of the Standing Committee of Hubei People's Congress (PSCH No.5), China.

## Author Contributions

XG, YB, and JL conceived and designed the study. LX, RS, SZha, XK, and CN collected the sample. XG, ZZ, and SZho executed the experiment and analyzed the samples. XG, CQ, and KL analyzed the data. XG and LX finished the first draft. MK revised the manuscript. All authors interpreted the data, critically revised the manuscript for important intellectual contents, and approved the final version.

## Funding

This study was supported by the Chinese Agricultural Research Systems (CARS-37) and the Key Research and Development Program of Tibet Autonomous Region (XZ202001ZY0044N).

## Conflict of Interest

The authors declare that the research was conducted in the absence of any commercial or financial relationships that could be construed as a potential conflict of interest.

## Publisher's Note

All claims expressed in this article are solely those of the authors and do not necessarily represent those of their affiliated organizations, or those of the publisher, the editors and the reviewers. Any product that may be evaluated in this article, or claim that may be made by its manufacturer, is not guaranteed or endorsed by the publisher.
